# Supplementary material for: Cryopreservation of infectious Cryptosporidiumparvum oocysts
Source: Nat Commun. 2018 Jul 23;9:2883. doi: 10.1038/s41467-018-05240-2 (PMC6056504; doi:10.1038/s41467-018-05240-2)
Supplement: Supplementary file 1 — Supplementary Information [file 41467_2018_5240_MOESM1_ESM.pdf]

Supplementary Information

Cryopreservation of Infectious *Cryptosporidium parvum* Oocysts

Jaskiewicz and Sandlin et al.

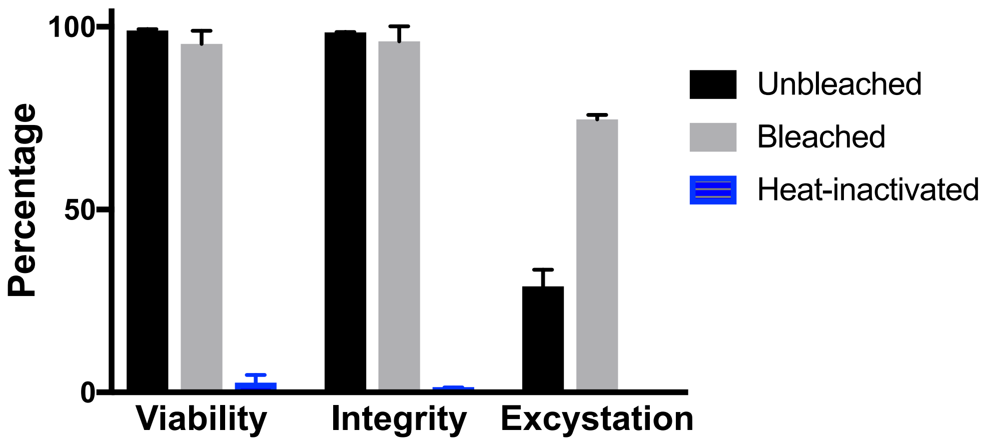

Supplementary Figure 1. Bleaching is a non-toxic method to permeabilize *C. parvum* oocysts. Viability, oocyst wall integrity and excystation rate were compared between bleached (5%, 7 min) and unbleached *C. parvum* oocysts in addition to a heat-inactivated control. Viability and integrity of the oocyst wall were measured by means of PI (10 µg/ml) and CFSE (5 µM) exclusion, respectively. Excystation rate was calculated as a percent of excysted oocysts after incubation with 0.75% taurocholic acid at 37°C (1 h). Values indicate means and error bars indicate standard deviation (n=3). While bleaching does not compromise viability and integrity of oocysts, it increases excystation rate.

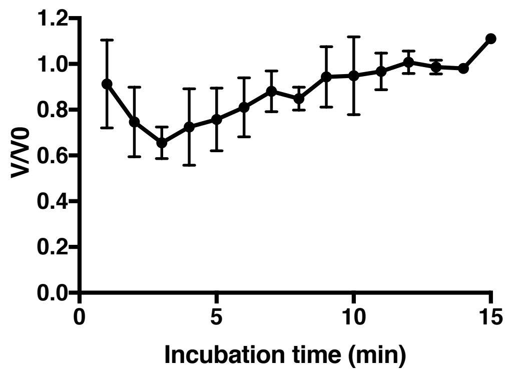

Supplementary Figure 2. Shrink-swell response of bleached *C. parvum* oocysts in DMSO. Volumetric changes of bleached oocysts exposed to 30% DMSO were measured by image analysis over 15 min of incubation. Loss of 34.4±6.9% of oocyst volume within 3 min, consistent with dehydration in the concentrated solution of DMSO, is followed by restoration of volume consistent with intracellular uptake of DMSO. Volume of DMSO treated oocysts (V) is compared to control oocysts in PBS (V0). Values indicate means and error bars indicate standard deviation (n=3).

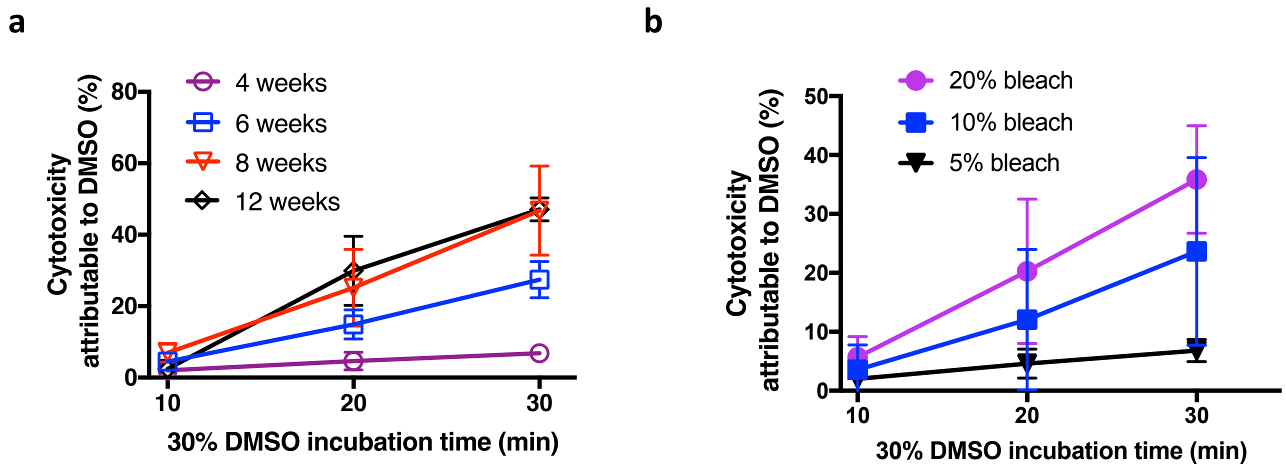

Supplementary Figure 3. Sources of variability in DMSO toxicity to *C. parvum* oocysts. (a) Bleached (5%, 1 min) and dehydrated (1 M trehalose, 10min) 4-, 6-, 8- and 12-week-old oocysts were treated with 30% DMSO, revealing that increased toxicity (based on PI inclusion) is a function of oocyst age (n=3). (b) Four-week-old *C. parvum* oocysts were bleached using 5, 10 or 20% concentration. Following dehydration in 1 M trehalose (10 min), oocysts were treated with a solution of DMSO to achieve a final concentration of 0.5 M trehalose/30% DMSO. Permeability of young oocysts increases as a function of bleach concentration used prior to trehalose/DMSO cocktail challenge. Values indicate mean toxicity and error bars indicate standard deviation (n=3).

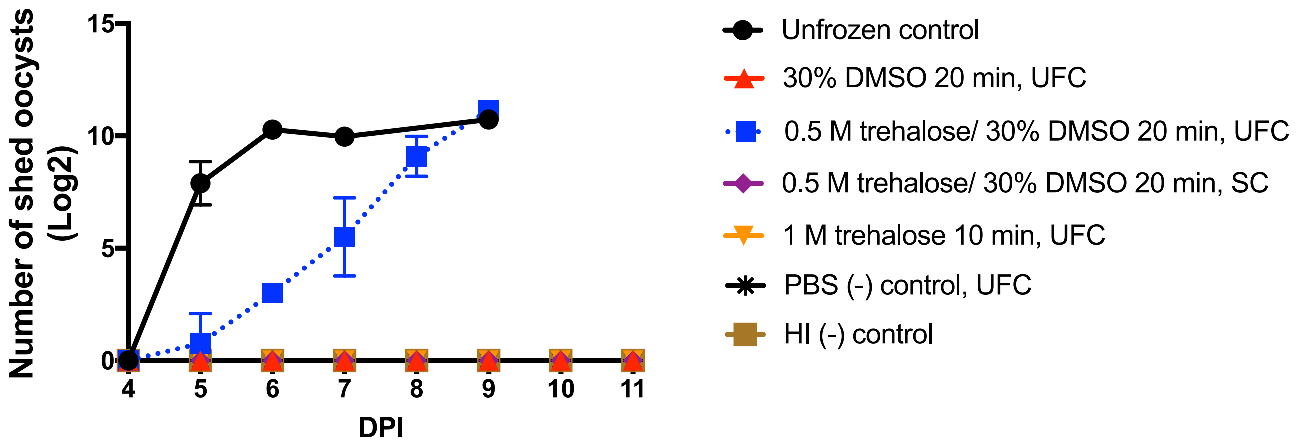

Supplementary Figure 4. Dehydration prior to DMSO treatment is necessary for cryopreservation of *C. parvum* oocysts using ultra-fast cooling. Bleached (5% bleach, 1 min) *C. parvum* oocysts were frozen using ultra-fast cooling (UFC) or slow cooling (SC) methods following treatment with 30% DMSO, with or without dehydration by trehalose. Positive (unfrozen) and negative controls (frozen in PBS or heat-inactivated) were included. "30% DMSO 20`, UFC", "PBS (-) control, UFC" and "HI (-) control" were performed twice. IFN-γ knockout mice (n=2-3) were inoculated orally with 5,000 PI^- oocysts. Intensity of fecal shedding was quantified daily by microscopic enumeration of oocysts in 30 fields of acid-fast stained fecal smears under 1000x magnification. Values indicate means of log transformed oocysts count and error bars indicate standard deviation. Both dehydration in trehalose and addition of 30% DMSO are necessary for cryopreservation using ultra-fast cooling. The identical cryopreservation protocol using slow cooling rates (1ºC/min), failed to produce infection in mice. One of 3 "PBS (-) control" mice became positive on DPI 17, which is consistent with an infectious dose smaller than 10 viable oocysts (*19*) and likely originated from contamination during thawing in the water bath.

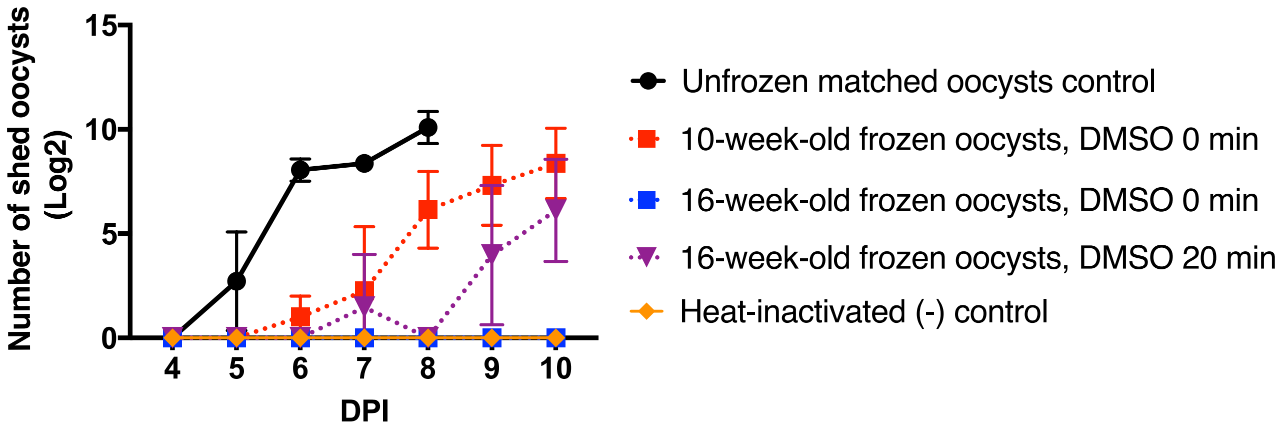

Supplementary Figure 5. Older oocysts require intracellular CPA for cryopreservation. Bleached (5%, 1 min) and dehydrated (1 M trehalose, 10min) *C. parvum* oocysts of different ages were frozen using the ultra-fast cooling protocol following 0 min or 20 min incubation in 0.5 M trehalose/30% DMSO solution. Positive (unfrozen) and negative controls (heat-inactivated) were included as matched controls. IFN-γ knockout mice (n=3) were inoculated orally with 5,000 PI^- oocysts. Intensity of fecal shedding was quantified daily by microscopic enumeration of oocysts in 30 fields of acid-fast stained fecal smears under 1000x magnification. Values indicate means of log transformed oocysts count and error bars indicate standard deviation. While 10-week-old oocysts frozen using 0 min protocol infected mice with the onset of shedding at 5 DPI, the same oocysts at 16 weeks of age were successfully cryopreserved only when using 20 min protocol, which allows for intracellular permeation of DMSO

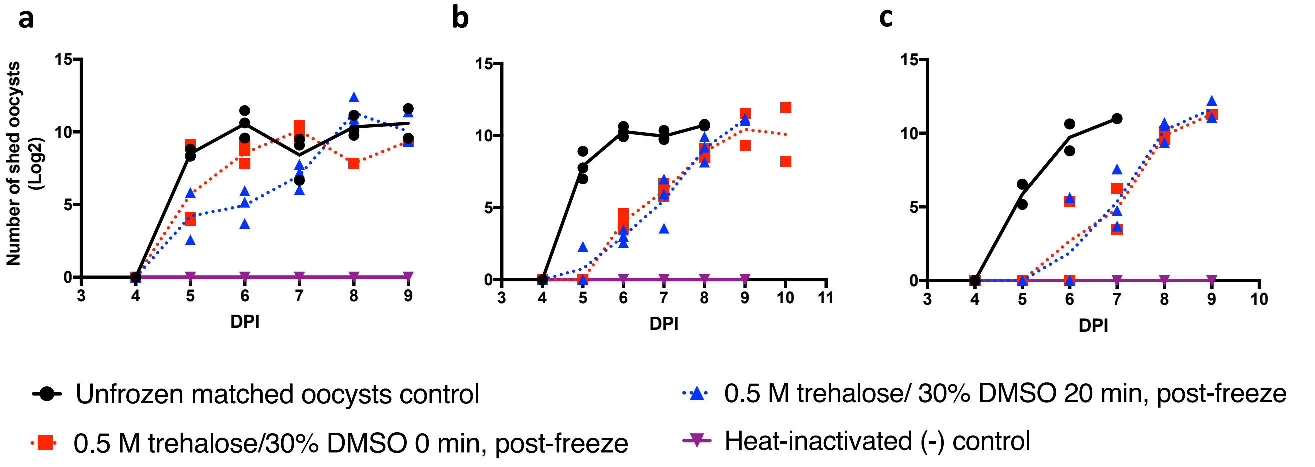

Supplementary Figure 6. Cryopreserved *C. parvum* oocysts are infectious to IFN-γ knockout mice (individual data plots). Dehydrated oocysts were vitrified using either the 0 min or 20 min DMSO incubation protocol. IFN-γ knockout mice were inoculated orally with 5,000 PI$^-$ thawed or unfrozen control oocysts. Intensity of fecal shedding was quantified daily by microscopic enumeration of oocysts in 30 fields of acid-fast stained fecal smears under 1000x magnification. Positive (unfrozen) and negative controls (heat-inactivated) were included as matched controls. To determine whether oocysts age has effect on the success of the cryopreservation protocol, (a) 1- (b) 6- and (c) 12-week-old oocysts were studied (n=2-3 mice). Values indicate a log transformed oocysts count from each mouse individually. Viability of oocysts was determined by PI exclusion prior to inoculation and was as follows: a) 43.1% and 76.7% b) 45.2% and 80.6% c) 53.1% and 72.4%, for 20 min and 0 min incubations, respectively.

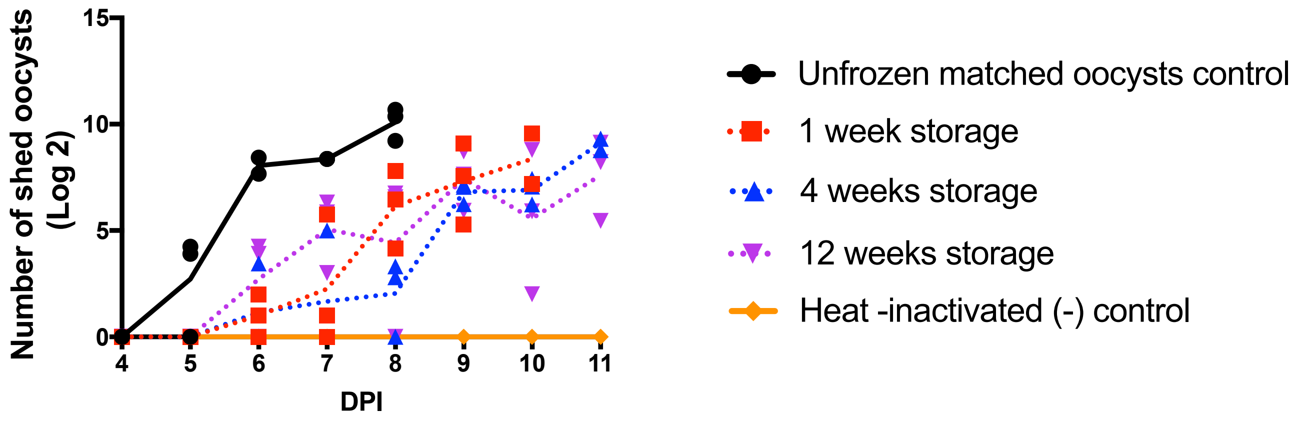

Supplementary Figure 7. Long-term cryogenic storage does not decrease infectivity of cryopreserved oocysts (individual data plot). Bleached *C. parvum* oocysts (5%, 1 min) were frozen using the ultra-fast cooling protocol following dehydration in trehalose (10 min) and 20 min incubation in 0.5 M trehalose/30% DMSO solution. Oocysts were stored in microcapillaries in liquid nitrogen and thawed after 1, 4 and 12 weeks. Positive (unfrozen) and negative controls (heat-inactivated) were included as matched controls. IFN-γ knockout mice were inoculated orally with 5,000 PI^- oocysts (n=3). Intensity of fecal shedding was quantified daily by microscopic enumeration of oocysts in 30 fields of acid-fast stained fecal smears under 1000x magnification. Values indicate a log transformed oocysts count for each individual mouse. Mice inoculated with cryopreserved oocysts developed similar infection at 6dpi regardless of the storage length, as evidenced by fecal shedding of oocysts. Viability of oocysts was determined by PI exclusion prior to inoculation and was as follows: 82.6%, 79.1% and 80% for 1 week, 1 month and 3 months of storage respectively.

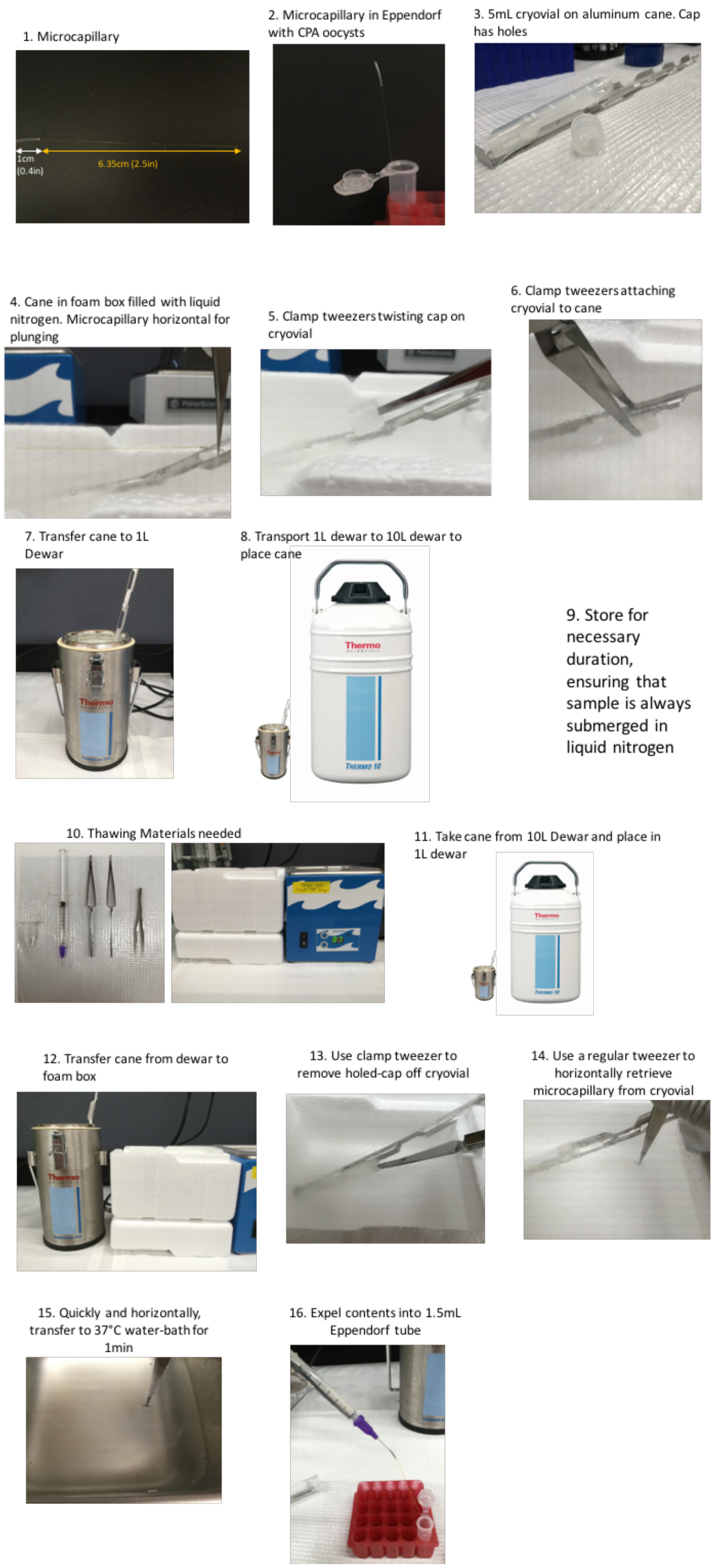

Supplementary Figure 8. Overview of critical steps and materials used for the cryopreservation protocol.

Explanatory variables	Coefficient (β)	SE	95% CI	*t*	p Value
Intercept	-0.96	0.239			
Age (weeks)	0.15	0.019	0.11 to 0.19	7.88	<0.001
DMSO incubation time (min)	0.14	0.007	0.13 to 0.16	18.46	<0.001
Bleach concentration (%)	0.09	0.010	0.06 to 0.11	8.40	<0.001
Total	N=236<br>R-squared=0.62<br>P<0.0001				

Supplementary Table 1. Sources of variability in DMSO toxicity to *C. parvum* oocysts. Regression table for multiple linear regression model explaining oocyst cytotoxicity attributable to DMSO (based on PI inclusion) with three explanatory variables: oocyst age, DMSO incubation time and bleach concentration. Coefficients are a measure of weight of the explanatory variable; SE, standard error; CI, confidence interval; *t*= t statistic calculated as ratio of regression coefficient to SE. All independent variables are statistically independent predictors of oocysts cytotoxicity at P<0.001. The multiple variables model explains 62% of variability in cytotoxicity between oocysts at P<0.0001.

C. parvum isolate	Oocysts age	Trehalose (0.5 M)	DMSO (30% v/v)	DMSO incubation time	Freezing method	Infectivity in animals after thawing
MD and Iowa	1-12 weeks	Yes	Yes	0 min	UFC	Yes
Iowa	16 weeks	Yes	Yes	0 min	UFC	No
MD and Iowa	1-16 weeks	Yes	Yes	20 min	UFC	Yes
Iowa	6 weeks	No	Yes	20 min	UFC	No
MD	1 week	Yes	No	-	UFC	No
MD and Iowa	1-16 weeks	No	No	-	UFC	No
MD	3 weeks	Yes	Yes	20 min	SC	No

Supplementary Table 2. Summary of cryopreservation protocols and their outcome in animal models. Presence of both trehalose and DMSO are essential for successful cryopreservation using ultra-fast cooling (UFC). While extracellular DMSO is sufficient to cryopreserve oocysts younger than 12 weeks of age, older oocysts require intracellular presence of DMSO. Slow cooling (SC) following treatment with trehalose and DMSO (20 min incubation), does not yield infectious oocysts.

Supplementary Methods.

Ultra-fast cooling of oocysts. The ultra-fast cooling method reported here was demonstrated to be a robust method to cryopreserve oocysts using either a 0 min or 20 min exposure to DMSO depending upon oocyst age. However, the method itself it extremely sensitive to fluctuations in technique. Thus, all experimentalists involved with this study were carefully trained prior to performing experiments with oocysts in order to master the technique. Below, we provide a more detailed protocol used for the long-term storage experiments and include potential missteps. Images of selected materials used in this study are included in Supplementary Figure 8. Occasionally, splashing may occur while using liquid nitrogen, so special attention to safety is critical, including but not limited to the use of face shield. Protocol:

1. Bleaching: 1 million oocysts (in 50 µl PBS contained in a 1.5 ml Eppendorf tube) of 10-week-old *C. parvum* (Iowa isolate) were chilled on ice then treated with 50µl of 10% Clorox bleach in PBS (5% final concentration) and incubated on ice for 1 min. The solution was then centrifuged at 18,000xg for 1 min. (Note: If a >500 µl volume is used, the centrifugation is increased to 2 min.) Supernatant was immediately removed and 400 µl of PBS added, followed by an additional 1 min centrifugation. This process is repeated three times to ensure bleach is removed from the solution. After the final washing step, the supernatant was completely removed in preparation for step #2.
2. The packed oocyst pellet was suspended in 10 µl of 1 M trehalose solution (prepared in PBS) and incubated at ambient temperature for 10 min.
3. 10 µl of a 60% v/v solution of DMSO (prepared in PBS) was then added to the oocysts to achieve a final concentration of 0.5 M trehalose/30% DMSO.
4. Oocysts were then immediately loaded into total of ten microcapillaries by capillary action. The exposure to DMSO was timed at 0 minutes in order to minimize DMSO permeation, however the

actual time of exposure varied for the ten capillaries, between 45 seconds and 3 minutes (despite the increased exposure period, it is unlikely that significant CPA permeation occurs based on data shown in Supplemental Figure 2). If desired, a second piece of Tygon tubing can be added to the opposite end of the microcapillary and heat sealed on both ends to ensure no leakage occurs.

5. Freezing: Tweezers were then used to pick up the microcapillary by the Tygon tubing. The capillary was then placed parallel to the liquid nitrogen (contained within a Styrofoam box). Using a steady but very rapid movement, the microcapillary was then plunged into the liquid nitrogen while maintaining a parallel position relative to the liquid surface. This ensures even heat transfer within the sample and is likely critical to successful cryopreservation outcome. Based on our experience with other applications, poor technique during the freezing and thawing steps are the most common reason for poor cryopreservation outcome.
6. The tweezers containing the microcapillary can rest on the bottom of the Styrofoam box while preparing for the next transfer step.
7. A cryocane (Thermo Fisher) containing an uncapped 5 ml cryovial (Globe Scientific) is then placed into the Styrofoam box and allowed to cool for 1-2 min.
8. The tweezers were then used to carefully maneuver the microcapillary into the cryovial, while ensuring that the sample stays submerged throughout. Even a brief exposure to air is likely to result in lethal ice crystallization.
9. Using a second set of tweezers, the perforated lid was carefully placed onto the cryovial to contain the microcapillary. The lid must be sufficiently perforated to allow gas exchange and prevent the cryovial from exploding due to gas pressure.
10. The cryocane was then submerged in a liquid nitrogen tank for long-term storage. The sample must remain submerged with liquid nitrogen at all times. If transport of cryocanes between bench and storage area is required, we recommend use of 1 L portable dewar.

11. Thawing: At the desired time, the cryocane was removed from storage and submerged in a Styrofoam box containing liquid nitrogen. Tweezers were used to carefully uncap the vial and remove the microcapillary.
12. The microcapillary was then adjusted so that it is parallel with the liquid surface.
13. Using a steady but rapid movement, the microcapillary was transferred into a 37ºC water bath and allowed to thaw for 1 minute.
14. A syringe filled with PBS and attached to the 30 g x1/2 needle (we recommend the of use needles with blunt end) was then used to eject the contents of the microcapillary into a 1.5 mL Eppendorf tube containing 500 µL of PBS. The syringe is pre-loaded with PBS to thoroughly wash out oocysts from the microcapillary. Approximately 100µl of PBS was used to expel contents and rinse contents of one capillary.
15. The solution was incubated in ambient temperature for 30 min to relieve the cryogenic stress.
16. The solution was then centrifuged at 18,000xg for 2 min.
17. The supernatant was then removed and the oocysts were resuspended in an appropriate amount of PBS, followed by evaluation of viability, excystation and infectivity.
